# Social interaction attenuates the extent of secondary neuronal damage following closed head injury in mice

**DOI:** 10.3389/fnbeh.2015.00275

**Published:** 2015-10-15

**Authors:** Vanessa M. Doulames, Meghan Vilcans, Sangmook Lee, Thomas B. Shea

**Affiliations:** ^1^Center for Neurobiology and Neurodegeneration Research, UMass LowellLowell, MA, USA; ^2^Biomedical and Biotechnology Program, University of Massachusetts LowellLowell, MA, USA; ^3^Department of Biological Sciences, University of Massachusetts LowellLowell, MA, USA

**Keywords:** Traumatic Brain Injury, neurodegeneration, cognitive impairment, oxidative damage, socialization, secondary injury

## Abstract

Recovery following Traumatic Brain Injury (TBI) can vary tremendously among individuals. Lifestyle following injury, including differential social interactions, may modulate the extent of secondary injury following TBI. To examine this possibility under controlled conditions, closed head injury (CHI) was induced in C57Bl6 mice using a standardized weight drop device after which mice were either housed in isolation or with their original cagemates (“socially-housed”) for 4 weeks. CHI transiently impaired novel object recognition (NOR) in both isolated and social mice, confirming physical and functional injury. By contrast, Y maze navigation was impaired in isolated but not social mice at 1–4 weeks post CHI. CHI increased excitotoxic signaling in hippocampal slices from all mice, which was transiently exacerbated by isolation at 2 weeks post CHI. CHI slightly increased reactive oxygen species and did not alter levels of amyloid beta (Abeta), total or phospho-tau, total or phosphorylated neurofilaments. CHI increased serum corticosterone in both groups, which was exacerbated by isolation. These findings support the hypothesis that socialization may attenuate secondary damage following TBI. In addition, a dominance hierarchy was noted among socially-housed mice, in which the most submissive mouse displayed indices of stress in the above analyses that were statistically identical to those observed for isolated mice. This latter finding underscores that the nature and extent of social interaction may need to vary among individuals to provide therapeutic benefit.

## Introduction

Traumatic Brain Injury (TBI) affects over 2 million Americans each year, resulting in >$70 billion in direct and indirect expenditures since 2000. The majority (>75%) of instances of TBI are classified as closed head injuries (CHI; Corso et al., 2006; Finkelstein et al., 2006; Faul et al., 2010).

TBI encompasses primary and secondary injury. Primary injury encompasses structural damage characterized by the stretching, compression, and tearing of blood vessels and tissue, the extent of which is dependent upon the nature of impact (Maas et al., 2008). Integrity of the white-matter tracts is compromised via shearing forces resulting in both axonal and myelin damage; the extent of this white-matter damage has been correlated with reduced cognitive function (Niogi et al., 2008). Focal contusions create swelling and bleeding within brain tissue and can lead to behavioral and psychological changes due to scarring (Maas et al., 2008). Compromised membrane and ion channel integrity is often reported (Maas et al., 2008). Secondary injury encompasses an often protracted cascade of biochemical processes that are instigated by primary injury, resulting in excitotoxicity, cellular energy deficits, inflammation, oxidative stress, and apoptosis (Xiong et al., 1997; Raghupathi et al., 2000; Yi and Hazell, 2006; Ansari et al., 2008; Cederberg and Siesjö, 2010). Secondary injury can accumulate over time and may not be detected for days to years following the primary injury (Gavett et al., 2010; Gilbert and Johnson, 2011). In many cases, such as military combat and concussions accompanying contact sports, primary injury may be mild enough not to warrant the hospitalization and medical care. In such instances, secondary injury can progress unchecked, often leading to neurodegeneration and cognitive dysfunction (Gavett et al., 2010; Borgens and Liu-Snyder, 2012; Glushakova et al., 2014).

Recovery following TBI varies tremendously. Many individuals do not improve following treatment but continue to decline due to secondary injury (Narayan et al., 2002). Secondary injury can progress unchecked in “mild” TBI (when there is no obvious initiating incident to warrant intervention). As such, secondary damage associated with mTBI can exceed that of more severe TBI. Timely intervention could potentially attenuate the degree of secondary injury.

Lifestyle may influence the consequences of secondary injury. A growing body of evidence supports the positive impact of socialization on other neurodegenerative conditions such as Alzheimer’s disease (Shea and Rogers, 2014; Shea et al., 2014). Maintenance of a large social network delays cognitive decline (Ertel et al., 2008). Even one-on-one interaction with institutional caregivers maintained cognitive performance for nursing home residents (van der Ploeg et al., 2013). Social isolation has been implicated in impaired memory and cognitive performance in normal and transgenic mice, alterations in in turnover and biosynthesis of dopamine in aged rats, increased oxidative damage, hypercortisolism, and exacerbation of acute and chronic inflammatory processes (Sapolsky et al., 1997; Miura et al., 2002; Huong et al., 2005; Eluvathingal et al., 2006; Raison et al., 2006; Huang et al., 2011; Möller et al., 2011; Sapolsky, 2011; Doulames et al., 2014). Since aspects of the neurodegeneration accompanying secondary injury parallel neurodegneration in other conditions such as Alzheimer’s and Parkinson’s disease (Mortimer et al., 1985; Taylor et al., 1999; Lye and Shores, 2000; Goldman et al., 2006; Chen et al., 2007), it remains possible that social interaction may also attenuate, or facilitate recovery from, secondary injury following TBI. While there is to date no compelling evidence for efficacy of non-pharmacological approaches (Meyer et al., 2010; Bowen et al., 2011), there is neither any evidence of efficacy for more conventional approaches (e.g., physio-and occupational therapy; Hellweg, 2012). Moreover, inclusion of social activity need not preclude any other therapeutic approaches. To test the hypothesis that social activity may curtail the extent of secondary damage following TBI, we examined the impact of housing of mice in groups or individually on cognitive function and biochemical parameters following experimentally-induced CHI. Our findings support the notion that social interaction may be beneficial as part of therapy following TBI.

## Materials and Methods

Normal C57B1/6J female mice, 9–12 months of age (30 in each of two experiments), obtained from Charles River, were housed in our facility for 7 days, after which they were subjected to CHI using a standardized weight drop device (Northeast Biomedical, Tyngsboro, MA) modeled according to Flierl et al. (2009). Briefly, mice were anesthetized with 3% isoflurane and oxygen at a rate of 0.5–1.0 liters per minute. The cranium was shaved, sterilized with povidone iodine followed by 70% ethanol and a 1 cm incision was made, exposing the coronal and sagittal sutures of the skull to allow reproducible location of CHI and to confirm lack of penetrating damage. The weighted rod of the device was aligned with upper left quadrant of the skull and allowed to free-fall from a height of 0.5 cm. Following impact, the incision was closed via wound closure clips and the mouse was allowed to recover on a heated water-circulating pad until normal behavior resumed. Analgesic (0.015% buprenorphine) was administered twice a day for 3 days following injury and weight, body condition, and behavior were monitored daily for up to 4 weeks. Following injury, half of these mice were housed socially with their original cage mates (2–3 mice per enclosure) or individually (*n* = 15 mice/housing condition in each of two experiments). An additional 10 mice housed socially (2 per cage) and not subjected to CHI were included to obtain baseline levels. Mice were observed daily.

Cognitive performance was quantified prior to (baseline), 24 h following CHI, then weekly for 1 month following CHI by the Novel Object Recognition (NOR) and by navigation of a standard Y Maze as previously described (Antunes and Biala, 2012; Lee et al., 2012; Doulames et al., 2014). The Y Maze Spontaneous Alternation Test (Y Maze) is a reward-free behavioral test used to assess exploration of a novel environment by a mouse, with any sort of performance compromise being indicative of cognitive dysfunction. The Y Maze (TSE Systems; Chesterfield, MO, USA) consists of 3 identical gray PVC arms mounted symmetrically (120° between arms) onto an equilateral triangular center compartment. Mice were gently placed in the center compartment of the maze and their movement was recorded for 5 min. Entry into an arm was defined by the mouse having all 4 feet within the arm. The pattern of exploration of the Y maze was analyzed over a 5 min interval. The frequency of visitation of the 3 arms of the maze in sequence (e.g., left, right, bottom, left, right, bottom, etc.) vs. total arm changes regardless of sequence was defined as the “percent alternation”. The total number of changes in arms over this 5 min interval was defined as an index of activity (Lee et al., 2012; Doulames et al., 2014). For NOR, each mouse was placed in a novel cage (arena) with 2 identical objects for 5 min (acclimation period), after which the mouse was removed, the arena cleaned (to remove any scent), one of the objects was replaced with a novel object, and the mouse was returned to the arena for an additional 5 min (test period). The amount of time the spent interacting with each object (e.g., touching, climbing, sniffing) was recorded. The test period was repeated 24 h later with the same original object but a different novel object. The above regimen (i.e., acclimation, followed by testing 5 min and 24 h after acclimation) was carried out at baseline, 24 h after CHI, and at weekly intervals for 1 month after CHI. Values represent the % of time interacting with the novel object vs. the total time interacting with either object (time with novel object/(time with novel object + time with familiar object). The pattern of exploration of the Y maze was analyzed over a 5 min interval. The frequency of visitation of the 3 arms of the maze in sequence (e.g., left, right, bottom, left, right, bottom, etc.) vs. total arm changes regardless of sequence was defined as the “percent alternation”. The total number of changes in arms over this 5 min interval was defined as an index of activity (Lee et al., 2012).

Housing, induction of CHI and cognitive testing were all carried out with the approval of our Institutional Animal Care and Use Committee. Following cognitive analyses, mice were sacrificed for biochemical, histological and electrophysiological analyses (also in accordance with our Institutional Animal Care and Use Committee) via CO_2_ asphyxiation and immediate decapitation; the same mice were used for all behavioral, biochemical and electrophysiological examination.

For monitoring of signaling, hippocampi were harvested and placed in oxygenated artificial cerebrospinal fluid (0.125 mM NaCl, 2.5 mM KCl, 0.1 mM MgCl_2_, 0.2 mM CaCl_2_, 0.125 mM NaH_2_PO_4_, 25 mM NaHCO_3_) supplemented with 100 mM sucrose (Serra et al., 2010). Sagittal slices (750 μM) cut with a Stoelting tissue slicer under sterile conditions were bathed in oxygenated artificial cerebrospinal fluid and transferred to the electrode recording area within a petri dish containing a 60-channel multielectrode array (MEA). Neuronal signaling was recorded over 30 s intervals using a DT9814 data acquisition system (Data Translation; Marlborough, MA, USA) and MEA-1060-INV amplifier (Multichannel Systems). Signals >4 mV in amplitude were quantified across all channels as previously described by Zemianek et al. (2012, (2013a,b).

Corticosterone concentration was measured via the Corticosterone Enzyme Assay Kit (Arbor Assays, Ann Arbor, MI, USA) according to the manufacturer’s instructions. Following sacrifice via CO_2_ asphyxiation, mice were exsanguinated via cardiac puncture. Blood was collected within the same 2 h period each morning to avoid potential variation due to circadian rhythm (e.g., Nelson et al., 1975). Blood samples were immediately allowed to clot on ice for 20 min prior to centrifugation (2000 × g at 4°C for 10 min). Aliquots (10 μl) of the resulting serum supernatant were immediately diluted 1:200 with the kit Assay buffer and incubated with a corticosterone-peroxidase conjugate, a corticosterone anti-sheep polyclonal antibody, and visualized by incubation with horseradish peroxidase at 450 nm.

Whole brains were homogenized with 20 strokes of a glass homogenizer and resuspended at 100 mg/mL in ice-cold phosphate-buffered saline (PBS; pH 7.4) containing 0.05% butylated hydroxytoluene as an antioxidant. Homogenates were derived from 3 mice for each condition, and each homogenate was assayed in duplicate. Homogenates were clarified by centrifugation (10,000 g at 4°C for 5 min) and the supernatants were frozen at −80°C until analysis. Total antioxidant capacity was determined in brain tissue homogenates via the OxiSelect Total Antioxidant Capacity Assay (Cell BioLabs, San Diego, CA, USA) to determine total antioxidant action. Following the manufacturer’s instructions, tissue was resuspended at 50 mg/mL in PBS and centrifuged (10,000 × g at 4°C for 10 min). Supernatants (10 μg/mL total protein) were combined with an equal volume of a proprietary assay mixture containing Cu^2+^ and a reagent which reacted with Cu^2+^ that was reduced by the residual antioxidants in brain homogenates, and “mM copper reducing equivalents” were quantified spectrophotometrically at 492 nm.

Aliquots of the above brain tissue homogenates were also analyzed by immunoblot analyses for amyloid beta (Abeta), phosphorylated and total tau and phosphorylated and total neurofilaments (Mortimer et al., 1985; Taylor et al., 1999; Meyer et al., 2010; Bowen et al., 2011). Homogenates were diluted to a final protein concentration of 5 mg/ml in 0.125mM Tris-HCl containing 1% Triton-X, 150 mM NaCl, 2 mM EDTA/EGTA, and a protease/phosphatase inhibitor cocktail. Samples were centrifuged (20 min at 12000 rpm at 4°C) and the resulting supernatants diluted in 0.125M Tris-HCl containing 4% SDS, 10% 2-mercaptoethanol, 20% glycerol, and 0.004% bromophenol blue, and boiled at 95°C for 5 min. Aliquots (40 μg total protein) were electrophoresed, and the separated proteins transferred to nitrocellulose membrane and blocked for 1 h with 5% non-fat milk in TBST (50 mM TrisHCl (pH 7.4) containing 150 mM NaCl and 0.1% Tween-20) at 4°C with shaking. Membranes were rinsed with TBST and incubated overnight at 4°C with 1:000 dilutions in TBST of PHF-1 (a mouse monoclonal directed against phosphorylated tau; Sigma Aldrich), Tau-5 (a mouse monoclonal antibody directed against tau regardless of phosphorylation state; LifeTechnologies), SMI-31 (a mouse monoclonal antibody directed against phosphorylated neurofilaments; Millipore) H31 (a rabbit polyclonal antibody generated in this laboratory against total neurofilaments regardless of phosphorylation state), DM1A (a mouse monoclonal antibody directed against α-tubulin) and a 1:2000 dilution of AB5078P (a rabbit polyclonal antibody directed against the 1–42 isoform of Abeta Millipore, Billerica, MA, USA), followed by rinsing 3X with TBST, incubation for 1 h at room temperature with anti-mouse or anti-rabbit alkaline phosphatase-conjugated secondary antibodies, and visualization of immunoreactive species via BCIP/NBT. Immunoreactive species were quantified using Image J software. AD is accompanied by an increase in phosphorylation of tau is hyperphosphorylated at serine residues 396 and 404; resultant phospho-isoforms are specifically are recognized by monoclonal antibody PHF-1 (Otvos et al., 1994). We therefore derived a ratio of PHF-1-reactive tau vs. total tau (as visualized by antibody Tau-5, which reacts with tau regardless of phosphorylation state). Assay of anti-tubulin via antibody DM1A was utilized as a loading control. Statistical comparisons of samples from three homogenates were carried out by ANOVA with *post hoc* analyses via Student’s *t* tests.

## Results

**Figure 1:**
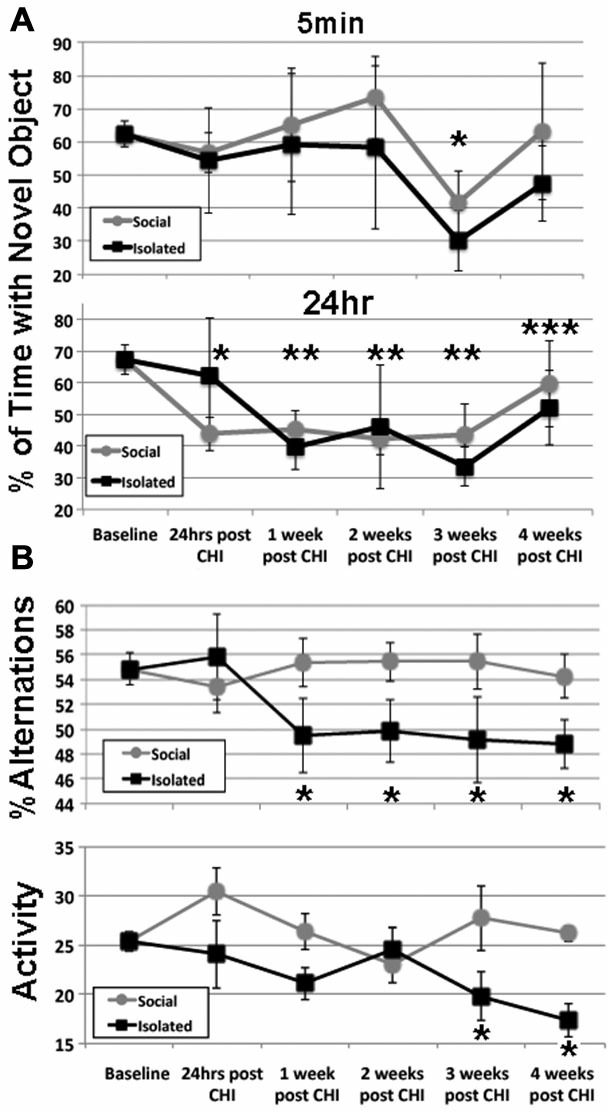
**Closed head injury (CHI) impaired cognitive performance; exacerbation by isolation. (A)** Presents at 5 min and 24 h after acclimation as indicated. Values represent the mean (± standard deviation) of the percentage of time devoted to the novel object (calculated according to the formula: seconds investigating novel object (seconds investigating novel object + seconds investigating familiar object). When tested 5 min after acclimation, both isolated and socially-housed mice displayed impaired performance at 3 weeks following CHI (asterisk; *p* < 0.07 and 0.01 vs. baseline for socially-housed and isolated mice, respectively. Both groups displayed novel object recognition (NOR) that did not differ from baseline at 4 weeks after CHI. When tested at 24 h after acclimation, socially-housed mice declined by 24 h after CHI (*p* < 0.05; single asterisk); isolated mice declined by an identical level by 1 week post CHI and both groups continued to display significantly impaired NOR until 3 weeks post CHI (*p* < 0.05; double asterisks). At 4 weeks post CHI, both social-housed and isolated mice displayed improvement in NOR (*p* < 0.07 and *p* = 0.05 vs. baseline, respectively; triple asterisks). **(B)** Presents the percent alteration and total activity as described in Materials and Methods. Socially-housed mice maintained performance in Y maze navigation identical to that at baseline for the entire 4 weeks following CHI. By contrast, mice housed under isolated conditions significantly declined in performance by 1 week following CHI, and continued to display significantly impaired performance for the entire 4 weeks (*p* < 0.05; asterisks). Isolated mice also displayed a significant (*p* < 0.05) decline in activity at 3 and 4 weeks following CHI as quantified by total changes in arms of the Y maze (*p* < 0.05; asterisks); socially-house mice maintained performance identical to that of baseline for the entire 4 weeks after CHI.

NOR following CHI was analyzed via NOR test at 5 min and 24 h after acclimation (Figure 1A). After 3 weeks post CHI, all mice displayed a transient decline in NOR 5 min after acclimation (Figure 1A). Isolated mice displayed a significant decline (*p* < 0.01), while socially-housed mice displayed only a trend towards significant decline (*p* < 0.07); NOR by both groups returned to levels statistically identical with baseline by 4 weeks. Over the first 3 weeks post CHI, all mice also displayed a significant decline in NOR at 24 h after acclimation, with a degree of improvement by 4 weeks post CHI (Figure 1).

Socially-housed mice maintained performance in Y maze navigation identical to that at baseline for the entire 4 weeks following CHI. By contrast, mice housed under isolated conditions significantly declined in performance by 1 week following CHI, and continued to display significantly impaired performance for the entire 4 weeks (Figure 1B). Isolated mice also displayed a significant (*p* < 0.05) decline in total activity at 3 and 4 weeks following CHI; socially-housed mice maintained performance identical to that of baseline for the entire 4 weeks after CHI (Figure 1B).

**Figure 2:**
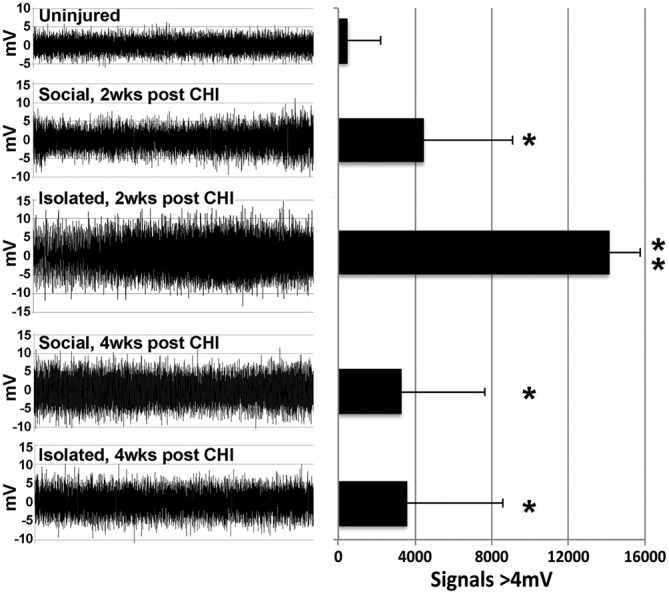
**CHI increased high amplitude signaling; isolation exacerbated this increase.** Panels present representative 30 s recordings of synaptic signaling in hippocampal slice from mice prior to (Baseline) and at 2 and 4 weeks following CHI. The accompanying graph presents quantification of signals >4 mV in amplitude. Values present the mean ± standard deviation of total signals >4 mV in amplitude quantified from one slice from each of 4 mice per condition. CHI significantly increased high amplitude signaling in all mice (*p* < 0.02; single asterisk). A transient further increase was noted for slices derived from isolated mice 2 weeks after CHI (*p* < 0.02 compared to all other conditions; double asterisk).

A significant (*p* < 0.02) increase in high amplitude (>4 mV) signals was observed in hippocampal slices at both 2 and 4 weeks following CHI vs. levels observed at baseline (Figure 2; *p* < 0.02). In addition, slices from mice maintained in isolation for 2 weeks following CHI displayed a further transient increase in high-amplitude signals (*p* < 0.02) compared to values at 4 weeks after CHI and to those observed from slices from socially-housed mice at 2 and 4 weeks after CHI (Figure 2).

**Figure 3:**
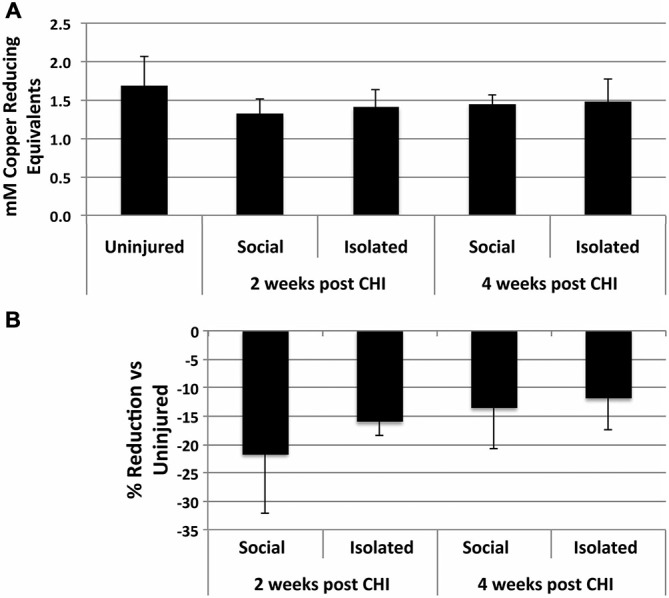
**CHI reduced total antioxidant capacity in brain tissue. (A)** Presents the mean antioxidant capacity ± standard deviation in mM copper reducing equivalents in for homogenates from brain tissue of uninjured mice and mice housed socially or individually for 2 and 4 weeks after CHI as indicated (*n* = 3 for each condition). **(B)** Presents the % reduction (± standard deviation) in antioxidant capacity for homogenates from brain tissue from mice subjected to CHI vs. uninjured mice. Mice subjected to CHI displayed decreases from 10–20%; these decreases did not differ significantly from those of mice not subjected to CHI (*p* = 0.26; ANOVA).

CHI induced a 10–20% reduction, not statistically significant) in antioxidant capacity in homogenates of brains from socially-housed and isolated mice at both 2 and 4 weeks after CHI capacity, indicating a minor induction of increased oxidative species following CHI (Figure 3).

**Figure 4:**
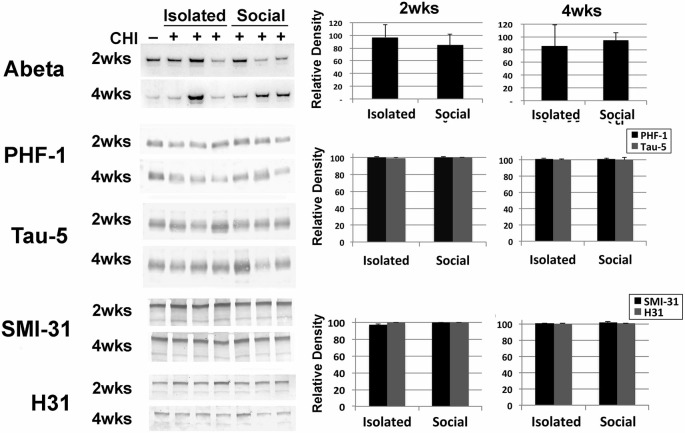
**CHI did not alter levels of Abeta, or levels or total or phosphorylated tau or, neurofilaments.** Panels present representative immunoblots of brain homogenates from uninjured mice and mice 2 and 4 weeks after CHI probed with anti-Abeta, PHF-1 and Tau-5 (directed against phospho-tau and total tau, respectively) and SMI-31 and H31 (directed against phospho-NF-H and total NF-H, respectively). The accompanying graphs present densitometric quantification; values represent the mean ± standard error from 3 homogenates (each assayed in duplicate) each from each condition as a percentage of levels observed prior to CHI. Additional immunoblots were probed with an antibody (DM1A) directed against alpha- tubulin as a loading control (not shown). Levels of these proteins were unchanged following CHI.

Brain tissue homogenates were analyzed for increases in Abeta and increases in total or phosphorylation of tau and neurofilaments as indices of neurodegeneration. No differences were detected following CHI for either socially-housed or isolated mice (Figure 4).

**Figure 5:**
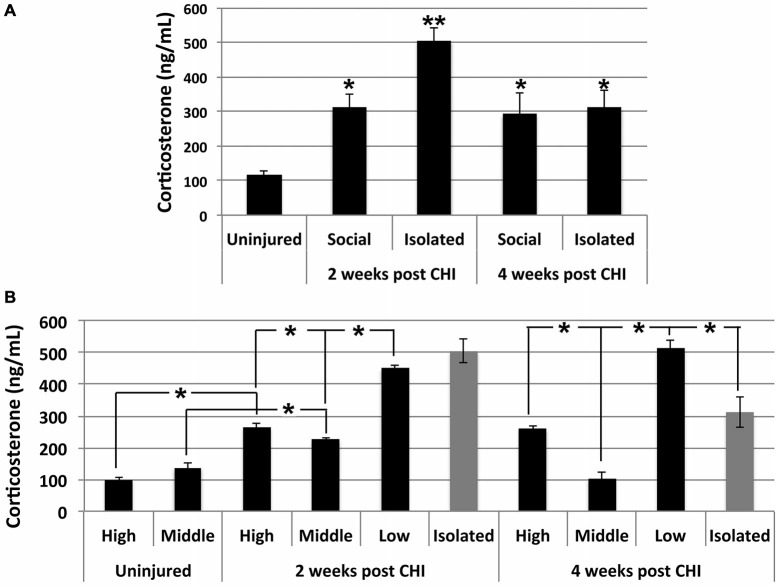
**CHI increased corticosterone levels in serum; exacerbation by isolation and social hierarchy. (A)** Serum corticosterone increased significantly in socially-housed and isolated mice at 2 and 4 weeks following CHI (*p* < 0.02; single asterisk). An additional significant increase was observed for isolated mice at 2 weeks following CHI vs. isolated mice at 4 weeks post CHI and socially-housed mice at both 2 and 4 weeks following CHI (*p* < 0.02; double asterisk). **(B)** Socially-housed mice were ranked according to the extent of aggressive behavior as high, middle and low as described in the text. High and low ranked mice subjected to CHI displayed significantly increased corticosterone vs. from uninjured high and low ranked mice, respectively (*p* < 0.02); note that uninjured mice were housed in duplicate, and therefore there is no middle rank for uninjured mice. Corticosterone levels in mice subjected to CHI were significantly higher in low rank mice than high and middle rank mice; levels in high rank mice were significantly higher than middle rank mice, and levels in middle ranked mice differed significantly from both highest and lowest ranked mice (*p* < 0.02). Low rank mice displayed corticosterone levels statistically identical to those of mice housed in isolation at 2 weeks and more elevated (*p* < 0.02); than those of mice housed in isolation at 4 weeks post CHI. Values represent the mean ± standard error from 3 homogenates (each assayed in duplicate) each from mice subjected to CHI and 2 homogenates each from mice not subjected to CHI (uninjured); asterisks indicate significant differences.

Significantly elevated levels of corticosterone were observed in serum in both socially-housed and isolated mice at 2 and 4 weeks after CHI vs. baseline levels (*p* < 0.02); a transient further significant increase was observed in isolated mice at 2 weeks after CHI vs. isolated mice at 4 weeks and socially-housed mice at both 2 and 4 weeks after CHI (*p* < 0.02; Figure 5A).

Corticosterone levels were influenced by social hierarchy (*p* < 0.02; Figure 5B). Daily observation allowed behavioral characterization of socially-housed mice (which were housed in groups of 3) as dominant (displaying the most aggressive behavior including barbering, territory marking, biting, and mounting), submissive (evidenced by extensive self-barbering, being attacked and/or mounted and exclusion from a nest fashioned by the other 2 mice), and a third category which was submissive to the dominant mouse, yet dominant over the submissive mouse. These categories were defined as high-, low-and middle-rank, respectively. Among socially-housed mice, the highest corticosterone levels were observed in low-rank mice, the lowest corticosterone levels were observed in middle ranked mice, and levels between these two were observed in high ranked mice; these differences were significant. High- and low-rank mice each displayed significantly elevated corticosterone levels at both 2 and 4 weeks following CHI vs. high- and low-rank mice not subjected to CHI (Figure 5B). Notably, the low-rank mice displayed corticosterone levels statistically identical to those of mice housed in isolation at 2 weeks post CHI, and significantly greater than those of mice housed in isolation at 4 weeks post CHI (Figure 5B).

## Discussion

Essential towards development of and effective treatment for TBI is curtailing secondary injury. Secondary injury can be particularly insidious in that mild TBI can remain undetected, and/or be sufficiently benign that an individual fails to seek treatment. In such instances, unchecked secondary damage could equal or surpass that accompanying severe TBI for which therapeutic intervention was initiated. Herein, we utilized the reductionist approach of reproducible, experimental CHI in mice in efforts to determine whether or not social interaction could influence secondary injury. We considered that monitoring mice within 24 h after injury, followed by weekly observations for 1 month, would afford insight into whether any impact of CHI on cognitive or physiological parameters was derived exclusively from primary and/or secondary injury. Along this line of reasoning, observation of trauma within 24 h would indicate that it may have been derived from primary injury; by contrast, a more protracted appearance and/or increase in the extent of trauma over one or more weeks following CHI would be consistent with contribution (at least in part) of secondary injury. Our reductionist approach, which utilized differential housing of mice following CHI, allowed us to examine whether or not socialization could impact the nature and extent of injury. Our findings demonstrated that this was indeed the case: both cognitive and physiological facets of secondary injury were affected by differential housing. In our prior studies (Doulames et al., 2014), isolation for 4 weeks without CHI induced a decline in cognitive performance as assayed by Y maze navigation; however this decline was not significant vs. performance by mice maintained under social housing (*p* = 0.26). Herein, the decline in Y maze performance for mice maintained under isolation conditions for 4 weeks after CHI was significantly reduced compared to that of socially housed mice receiving CHI (*p* = 0.02; Student’s *t* test). These comparisons suggest that CHI impaired cognitive performance beyond that of isolation alone, and that housing under social conditions was capable of preventing the extent of decline induced by the combination of CHI followed by isolation. Notably, it remains unclear whether social interaction alleviated the progression of secondary injury, or instead whether isolation exacerbated secondary injury, or whether or not the differences observed between groups was derived in part by both.

Cognitive performance following CHI was affected by both primary and secondary injury, and furthermore was modulated by differential housing following CHI. CHI impaired both short- and long-term recognition memory as assayed by NOR at 5 min and 24 h after CHI, respectfully. Short-term memory declined in mice transiently at 3 weeks after CHI for mice housed socially or in isolation, suggesting that this transient decline was due to secondary injury and was not affected by social interactions. Long-term memory following CHI displayed a more complex decline, in which socially-housed mice declined within 24 h but mice maintained in isolation instead declined 1 week following CHI. Of interest would have been to assay NOR at 36, 48 and/or 72 h to determine more precisely the point at which isolated mice declined in long-term memory. Both groups continued to display impaired long-term memory until at least 3 weeks post CHI, and had substantially recovered in NOR by the 4th week after CHI, at which point their performance was nearly restored to that at baseline.

In contrast to NOR, Y maze navigation, which relies on spatial memory, was not impaired until 1 week following CHI, and moreover, was only impaired in mice housed in isolation. This protracted decline, coupled with its observation only for mice housed in isolation, suggested that impairment in spatial memory was not due to primary injury. Moreover, prevention of this decline in socially-housed mice holds the promise that social interaction may be a useful part of a therapeutic approach to curtail secondary injury following TBI. Also in contrast to NOR, mice maintained in isolation did not recover in Y maze navigation. Of interest would be to monitor mice for longer intervals to determine whether or not there was a more protracted recovery. Nevertheless, the observation of recovery in NOR suggests that CHI utilized herein did not induce significant permanent loss of neuronal function. This is consistent with prior studies demonstrating memory impairment following mild TBI without substantial neuronal death (Yarnell and Lynch, 1970; Lyeth et al., 1990; Miyazaki et al., 1992; Cacioppo and Hawkley, 2009).

CHI significantly increased high amplitude signaling in hippocampal slices, which was transiently exacerbated by isolation at 2 weeks but not 4 weeks post CHI. This finding is consistent with the association of excitotoxicity with secondary injury following TBI (Palmer et al., 1993; Brown et al., 1998; Rao et al., 1998; Yi and Hazell, 2006), and may in part underlie impaired cognitive performance.

Consistent with prior studies with rodents (Awasthi et al., 1997; Tyurin et al., 2000; Ansari et al., 2008) and with clinical findings (Bayir et al., 2002), we observed a reduction in total antioxidant capacity following CHI, indicative of increased oxidative stress. No differences in total antioxidant capacity were observed between socially-housed and isolated mice. Of interest would be to quantify reactive oxygen species and overall levels of oxidized protein and lipids.

Corticosterone levels were monitored as an index of overall stress (Levine and Treiman, 1964; Goodman et al., 1996). CHI significantly increased corticosterone, and this increase was transiently exacerbated by isolation at 2 weeks post CHI. Social hierarchy also exacerbated corticosterone levels following CHI. High-rank mice within their respective groups of 3 displayed aggressive behavior including barbering, territory marking, biting, and mounting both middle-rank and low-rank mice. Middle-rank mice also displayed dominant behavior over low-rank mice. Consistent with prior studies (Louch and Higginbotham, 1967; Schuhr, 1987; Sapolsky, 1992, 2005, 2011; Sapolsky et al., 1997), corticosterone levels were observed in decreasing order as low-rank mice > high-rank mice > middle-rank mice. Although observation of elevated corticosterone in high-rank vs. middle-rank mice may seem counterintuitive, high-rank mice undergo relatively more stress than middle-rank mice in order to maintain their dominance. Of interest was that low-rank mice displayed corticosterone levels identical to those of isolated mice at 2 weeks post CHI, and significant higher than those of isolated mice by 4 weeks post CHI. Extrapolation of these findings to humans leaves open the possibility that the nature and extent of social interactions will likely differ among individuals; while social interaction may indeed form an important part of a therapeutic program following TBI, no single form of social interaction will be appropriate for all individuals. For example, group meetings may be appropriate for some individuals (Ertel et al., 2008), while, conversely, limiting interactions to relatively brief one-on-one conversations with a therapist may be more appropriate for others (van der Ploeg et al., 2013).

A limitation of our study is that we did not sacrifice mice immediately following CHI for monitoring of signaling or biochemical analyses, which would have allowed us to determine whether or not the increases in oxidative species, excitotoxic signaling and corticosterone accompanied primary injury or instead accumulated by week 2 post CHI as a consequence of secondary injury, or both. However, since excitotoxic signaling and corticosterone underwent transient exacerbation in isolated mice, it is likely that these deleterious effects were at the very least enhanced by secondary injury; had they been entirely due to primary injury, both groups of mice would have been equally affected. Of interest would be to group 2 or 3 of the low-rank or middle-rank mice together, to determine whether or not this would alleviate corticosterone elevation following CHI, or whether a new dominance hierarchy would emerge. Similarly, grouping isolated mice after, e.g., 1 or 2 weeks may allow reversion of some of the impact of isolation following CHI.

No changes levels of Abeta, or levels and/or phosphorylation of tau or neurofilaments were observed following CHI as administered herein. This finding is consistent with our observation of cognitive recovery, but contrasts with studies demonstrating that even a single mild TBI can fosters alterations in these neurodegenerative hallmarks (Smith et al., 1999; Schmidt et al., 2001; Franz et al., 2003; McKee et al., 2009; Gavett et al., 2010; Johnson et al., 2012). Monitoring mice for a longer interval, post CHI, especially those maintained in isolation, may reveal protracted accumulation of Abeta or cytoskeletal abnormalities; however, other studies show can see increased phosphorylation of tau within 24 h and lasts up to 30 days (Huber et al., 2013). Methods for modeling TBI vary considerably; one possibility is therefore that CHI as administered herein was milder than that used in prior studies. A stronger CHI could be administered, but perhaps of more interest would be to administer repetitive CHI under the conditions utilized herein, to model repetitive injury that can accompany contact sports.

The various deleterious consequences observed herein following CHI are likely to be inter-related at least in part. For example, excessive corticosterone exposure can exacerbate excitotoxicity (Goodman et al., 1996), which may be reflected by our observation of high amplitude signals in hippocampal slices from mice 2 weeks post CHI. Hippocampal signaling abnormalities are anticipated to be reflected by impairment in memory. Chronic stress, reflected herein by increased corticosterone, impairs memory (Pavlides et al., 1993; McEwen, 1999). Oxidative stress both induces excitotoxicity and impairs memory (Daulatzai, 2013; Rachmany et al., 2013). These events likely occur in a deleterious feedback mechanism, since, for example, oxidative stress induces anxiety, which can further elevate corticosterone (Salim et al., 2011). Our demonstration that social isolation impairs cognitive performance, increases stress, and exacerbates excitotoxicity supports the notion that pro-active approaches including social interaction may influence the rate and extent of recovery following TBI.

Environmental enrichment has been shown to boost cognitive performance in mice not subjected to CHI, using the same cognitive tests as utilized herein, including augmenting the impact of social interaction (Doulames et al., 2014). Of interest would be to examine whether or not environmental enrichment, with and without housing in groups vs. isolation, would be effective following CHI.

## Conflict of Interest Statement

The authors declare that the research was conducted in the absence of any commercial or financial relationships that could be construed as a potential conflict of interest.
